# Usefulness of K-line in predicting prognosis of laminoplasty for cervical spondylotic myelopathy

**DOI:** 10.1186/s12891-023-06214-2

**Published:** 2023-02-11

**Authors:** Terumasa Ikeda, Hiroshi Miyamoto, Masao Akagi

**Affiliations:** Department of Orthopedic Surgery, Kindai University Hospital, Ohno-Higashi, Osaka-Sayama, Japan

**Keywords:** Cervical spondylotic myelopathy, K-line, Cervical spinal kyphosis

## Abstract

**Background:**

K-line is widely recognized as a useful index for evaluating cervical alignment and the size of the cervical ossification at the posterior longitudinal ligament (OPLL). The purpose of this study was to investigate whether the K-line could be a useful clinical tool for predicting the prognosis of laminoplasty (LP) for cervical spondylotic myelopathy (CSM).

**Methods:**

Adult CSM patients scheduled for cervical LP were recruited for this study. C2-7 angle, local kyphosis angle, and K-line was evaluated by T2-weighted sagittal magnetic resonance imaging (MRI). Clinical findings were evaluated by the JOA score and the recovery rate. Clinical and radiological findings were evaluated preoperation and final follow-up. Patients were grouped into K-line ( +) and K-line (-). Patients with Kline (-) were further divided into two sub-groups: disc type (anterior cord compression due to disc protrusion with kyphosis) and osseous type (due to osseous structure such as osteophyte).

**Results:**

Sixty-eight patients were included in the analysis. The recovery rate of K-line (-) group (*n* = 11,19.4%) was significantly worse than that of K-line ( +) group (*n* = 57, 50.6%,* p<0.05*). Among 11 K-line (-) patients, 7 were disc type and 4 were osseous type. Over the period of follow-up, the disc type K-line (-) patients changed to K-line ( +) and showed significantly better recovery rate (27.6%) compared to the osseous type K-line (-) group (5.0%, *p* < 0.05).

**Conclusion:**

The present of this study indicate that K-line may have a predictive value for clinical outcome in patients undergoing LP for CSM. K-line (-) of osseous type was worse than k-line (-) of disc type.

## Background

Laminoplasty (LP) is occasionally indicated for cervical myelopathy caused by a narrow spinal canal due to spondylosis and/or ossification of the posterior longitudinal ligament (OPLL). This procedure enables sufficient decompression in multi-segmental stenotic myelopathy. The effects of decompression with LP are attributed to two mechanisms, i.e., direct posterior decompression and posterior shifting of the spinal cord from anterior compressive lesions [1–3]. However, a large anterior bulge, as with a protruded disc or OPLL, often worsens the postoperative neurological recovery rate after LP [4–9]. Cervical kyphosis may also lead to poor surgical outcomes by interfering with posterior shifting of the spinal cord [3, 10]. Iwasaki et al. [4] reported that the neurologic outcome of LP for cervical OPLL was poor or fair in patients with occupying ratio greater than 60%, hill-shaped ossification, and postoperative kyphotic change in cervical alignment.

Fujiyoshi et al. [11] developed the K-line, a straight line connecting the midpoints of the spinal canal at C2 and C7 on plain lateral radiograph, as a new index to evaluate cervical alignment and OPLL size in one parameter, i.e., OPLL did not exceed the K-line in the K-line ( +) group and did exceed the K-line in the K-line (-) group.

With regard to cervical spondylotic myelopathy (CSM), Suda et al. [10] reported that the patients with local kyphosis≧13 degrees exhibited poorer clinical outcomes than those without kyphosis due to the mechanisms described above. On the other hand, Chiba et al. [12] reported that several patients with cervical kyphosis obtained an acceptable clinical outcome after LP alone, probably because of the slackening of the spinal cord due to reduced multilevel disc height, which would not be compatible with cervical OPLL. Therefore, usefulness of K-line for CSM was uncertain. The purpose of the present study was to investigate whether K-line can provide a predictor of the clinical outcomes of LP for CSM.

## Methods

Participants aged between 32 and 92 were recruited for this study (mean age 60.3 years). Inclusion criteria were patients who underwent primarily LP for CSM at Kindai University hospital. Exclusion criteria were myelopathy caused by single-level disc herniation, OPLL, or a history of cervical spinal surgery, spinal tumor, trauma, and infection. This study was conducted after the protocol had been approved by the Institutional Review Board of Kindai University Hospital (Control Cohort Study, No.2020–025), and an informed consent was obtained from all patients.

### Study design

Two surgical decompression techniques were used 1) Miyazaki and Kirita’s method　(*N* = 64), and 2) a modified version of Kurokawa’s method (*N* = 4). All patients were followed-up for at least two years to obtain clinical outcomes and radiological evaluations. Patients were grouped into K-line ( +) and K-line (-). Patients with K-line (-) were further divided into two sub-groups: disc type (anterior cord compression due to disc protrusion with kyphosis) and osseous type (due to osseous structures and local kyphotic alignment).

### Surgical technique

The method of Miyazaki and Kirita was a procedure in which bilateral gutters were made and the laminae were split in the middle with a high-speed drill. The laminae were kept open with nylon sutures in the deep fascia bilaterally [13]. The Kurokawa’s method was the procedure in which mid-splitting of the spinous processes was performed using a T-saw. And then, the bone harvested from the iliac bone was fixed between the opened spinous process with a wire [14]. The modified Kurokawa’s method used hydroxyappatie spacers instead of the iliac bone. Both procedures were performed by two spine specialists (H.M. and T.I.).

### Clinical evaluation

The Japanese Orthopaedic Association (JOA) scoring system was used to evaluate the severity of cervical myelopathy preoperatively and at the final follow-up. Using the JOA score, the recovery rate (RR) was calculated as previously described [15]:

RR (%) = (postoperative JOA score – preoperative JOA score) / (17- preoperative JOA score) × 100.

### Radiological evaluation

All radiological evaluations were made on T2- weighted sagittal images of MRI, and preoperative findings and findings at the time of final follow-up were recorded. C2-7 lordotic angle was measured in all cases. In CSM patients, although C2-7 angle was positive, local kyphosis existed due to the malalignment (e.g., sigmoid, reverse sigmoid, and kyphosis) in several patients. In those cases, local kyphosis angle was also measured. The K-line, a straight line connecting the midpoints of the spinal canal at C2 and C7 level, was drawn. The patients were divided into two groups; K-line (-) and K-line ( +). In the K-line (-) group, the anterior structural factors (e.,g., disc herniation, osteophyte, or local kyphotic beak of the vertebra) crossed the K-line with no space between the K-line and the anterior wall of the canal. On the other hand, in the K-line ( +) group, the anterior structures did not exceed K-line and stayed within the ventral area of K-line. K-line (-) patients were further grouped into disc type and osseous type.

### Statistical analysis

Clinical parameters such as gender, age, preoperative JOA score, the JOA score at the follow-up, RR at the time of final follow-up, and preoperative and postoperative (at final follow-up) radiological parameters such as C2-7 angle and the local kyphotic angle were compared between K-line (-) and K-line ( +) group.

Statistical analyses of the data were performed using Stat Flex Ver.6 (Artech Co.,Ltd.,Osaka, Japan). The results were presented as means ± SD. The degree of freedom was calculated using Wilcoxon test and Mann–Whitney U test with *p* < 0.05 considered statistically significant.

## Results

Sixty-eight patients (50 males, 18 females; mean age at point of surgery (± SD) 60.3 ± 12.4) undergoing cervical LP decompression surgery for CSM were investigated. The mean follow-up period was 74.2 months. Participant characteristics and pre-surgical and post-surgical clinical and radiological findings were provided in Table 1.

**Table 1:** Background characteristics of the participants

Characteristic	
Age (years old)	60.3 ± 12.4
Sex (male/female)	50 / 18
Follow-up period (months)	74.2 ± 43.5
The JOA at Pre-op (points)	9.7 ± 2.9
The JOA at FU (points)	13.0 ± 2.6
The RR (%) of the JOA	45.5 ± 28.0
C2-7 angle at Pre-op (degrees)	4.1 ± 12.5
C2-7 angle at FU (degrees)	6.6 ± 14.3

Data are presented as means ± SD

*JOA* Japanese orthopaedic association score

*RR* Recovery rate

*Pre-op* Pre-operation

*FU* Final follow-up

### Surgery and K-line group allocation

The spinal levels of decompression were from C3 to C7 in 47 patients, C3 to C6 in 8 patients, C4 to C7 in 4 patients, C2 to C7 and C3 to Th1 in 3 patients each, and C3 to C5, C4 to C5 and C4 to Th1 in one patient each. The operative technique was performed the Miyazaki and Kirita’s method and a modification of Kurokawa’s method. Eleven patients were found with K-line (-) (8 male, 3 females; mean age 57.2 ± 17.5, and 57 patients were found with K-line ( +) (42 male, 15 females; mean age 60.9 ± 11.0 Table 2). Eleven patients with K-line (-) were further divided into two sub-group; disc type (anterior cord compression due to disc protrusion with kyphosis) 7 patients (mean age 57.4 ± 15.1) and osseous type (due to osseous structures with kyphosis) 4 patients (mean age 56.8 ± 23.9).

**Table 2:** Comparison between K-line (-) and K-line ( +)

	K-line(-)	K-line( +)	p
Numbers of the patients	11	57	
Age (years old)	57.2 ± 17.5	60.9 ± 11.0	NS
The JOA at Pre-op (points)	10.1 ± 4.3	9.7 ± 2.6	NS
The JOA at FU (points)	11.6 ± 4.1	13.3 ± 2.2	< 0.01
The RR of the JOA (%)	19.4 ± 25.2	50.6 ± 25.8	< 0.01
C2-7angle at Pre-op (degrees)	-10.1 ± 9.0	6.8 ± 11.2	< 0.01
C2-7angle at FU (degrees)	-11.8 ± 10.4	10.2 ± 12.1	< 0.01
Local kyphosis angle at Pre-op (degree)	16.6 ± 10.0	1.6 ± 3.1	< 0.01
Local kyphosis angle at FU (degree)	14.7 ± 7.6	1.3 ± 3.1	< 0.01

Data are presented as means ± SD

*JOA* Japanese orthopaedic association score

*RR* Recovery rate

*Pre-op* Pre-operation

*FU* Final follow-up

### Clinical findings

Overall, the difference in mean JOA score at pre-operation and at final follow-up was statistically significant; pre-surgery JOA = 9.7 (± 2.9) (range, 1–14 points) vs. post-surgery JOA = 13.0 (± 2.6) (range, 1–17 points) [Wilcoxon test, *P* < 0.01]. The Mean RR of the JOA score was 45.5%. The difference between the pre-operation JOA score and JOA score at the time of final follow-up was 10.1 ± 4.3 and 11.6 ± 4.1 in the K-line (-) group and 9.7 ± 2.6 and 13.3 ± 2.2 in K-line ( +) group, respectively. The JOA score at final follow-up was the significant difference between K-line ( +) and K-line (-) [Mann–Whitney U test, *P* < 0.01; Table2]. There was significant difference of the mean RR between the K-line (-) group (19.4%) and the K-line ( +) group (50.6%) [Mann–Whitney U test, *P* < 0.01; Table2]. K-line (-) were further divided into two sub-group; disc type (*N* = 7) and osseous type (*N* = 4). We compared the clinical data between them. The pre-operation JOA score and JOA score at the time of final follow-up was 11.1 ± 3.1 and 13.4 ± 1.6 in disc type and 8.1 ± 5.8 and 8.6 ± 5.8 in osseous type, respectively. That is, the JOA score at final follow-up was not the significant difference between disc type and osseous type. The mean RR of K-line (-) disc type was 27.6% at final follow-up and that of in K-line (-) osseous type was only 5.0% [Mann–Whitney U test, *P* < 0.05].

### Radiological findings

Overall, the C2-7 angles at preoperatively and at follow-up were 4.1 ± 12.5 and 6.6 ± 14.3 degrees respectively (Table 1). Preoperative C2-7 angle was significantly smaller in the K-line (-) group (-10.1 ± 9.0 degrees) than in the K-line ( +) group (6.8 ± 11.2 degrees) [Mann–Whitney U test, *P* < 0.01; Table2]. In addition, the C2-7 angle at final follow-up was significantly smaller in the K-line (-) group (-11.8 ± 10.4 degrees) compared with the K-line ( +) group (10.2 ± 12.1 degrees) [Mann–Whitney U test, *P* < 0.01; Table2]. Preoperative local kyphosis angle was also significantly larger in the K-line (-) group (16.6 ± 10.0 degrees) compared with the K-line ( +) group (1.6 ± 3.1 degrees) [Mann–Whitney U test, *P* < 0.01; Table2]. Furthermore, local kyphosis angle at follow-up was significantly larger in the K-line (-) group (14.7 ± 7.6 degrees) than in the K-line ( +) group (1.3 ± 3.1 degrees) [Mann–Whitney U test, *P* < 0.01; Table2]. Preoperative C2-7 angle was significantly smaller in osseous type of K-line (-) (-17.3 ± 7.1 degrees) than disc type of K-line (-) (-6.0 ± 7.4 degrees) [Mann–Whitney U test, *P* < 0.05]. The C2-7 angle at final follow-up was significantly smaller in osseous type (-20.8 ± 12.3 degrees) compared with disc type (-6.7 ± 4.5 degrees) [Mann–Whitney U test, *P* < 0.05]. Preoperative local kyphosis angle was osseous type (16.0 ± 5.7 degrees) and disc type (15.3 ± 12.4 degrees), respectively. Furthermore, local kyphosis angle at follow-up was osseous type (17.3 ± 2.4 degrees) and disc type (11.7 ± 7.8 degrees), respectively. The local kyphosis angle was not statistically difference at pre-operation and at time of final follow-up.

### Post-surgical K-line changes

**Fig. 1:**
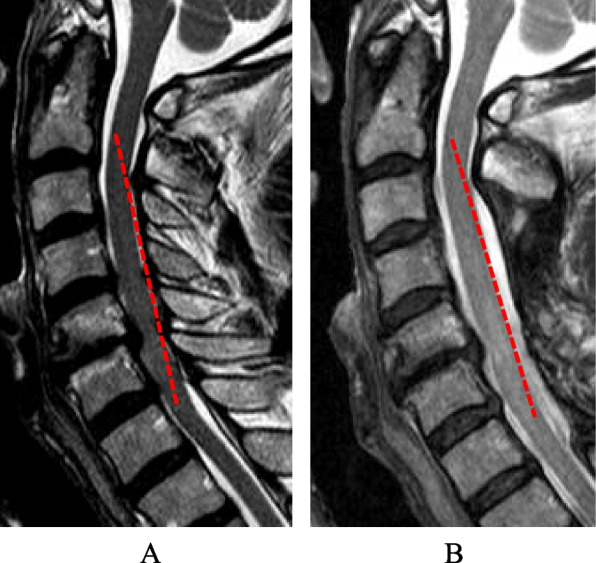
K-line ( +) case. Laminoplasty was performed from C3 to C7. C2/C7 angle was 29° at preoperation (**A**) and 28° at follow-up (**B**). The JOA score improved from 13 points to 15.5 points. The recovery rate of the JOA score was 62.5%

**Fig. 2:**
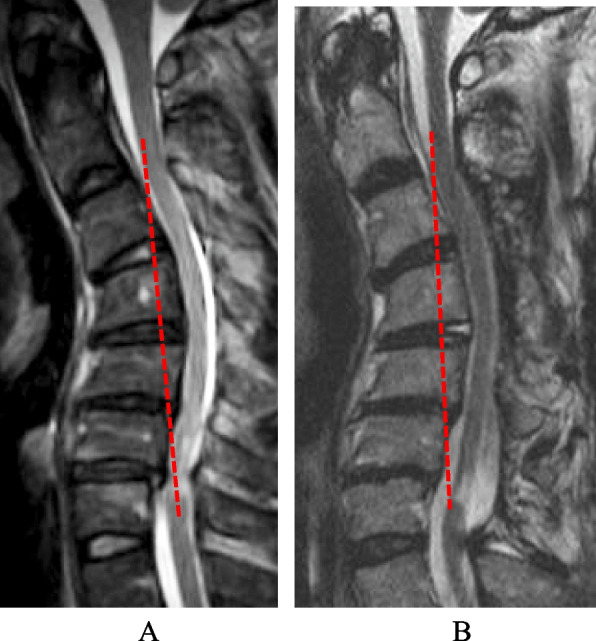
K-line (-) case. Laminoplasty was performed from C3 to C7. C2/C7 angle was -39°at preoperation (**A**) and -39° at follow-up (**B**). The JOA score improved from 7 to 9 points. The recovery rate of the JOA was 20%

**Fig. 3:**
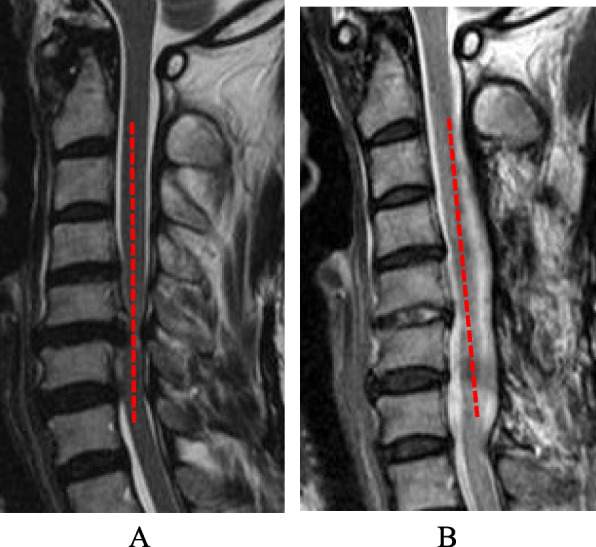
K-line (-) case. Laminoplasty was performed from C3 to C7. C2/C7 angle was -1° at preoperation (**A**) and -16° at follow-up (**B**). The JOA improved from 11 points to 13.5 points. The recovery rate of the JOA score was 41.7%. The protruded disc at C5/6 had a contact with K-line at preoperation (**A**). However, at the follow-up (**B**), the disc was absorbed, and the classification was changed to K-line ( +)

Representative MRIs of preoperative K-line ( +), osseous type, and disc type are provided in Figs. 1, 2 and 3, respectively. At follow-up, we found that 6/7 cases of preoperative K-line (-) changed to K-line ( +) during follow-ups because of absorption of the protruded disc (Fig. 3). On the other hand, 4 cases of osseous type that was preoperative K-line (-) remained as K-line (-) at follow-up (Fig. 2).

## Discussion

The results of our study indicate that the K-line, which is typically used to predict the clinical outcome of LP in OPLL, may also be useful in CSM. Interestingly, cases preoperatively defined as K-line (-) showed significantly poorer clinical outcome after LP compared to those with K-line ( +). The surgical outcomes for CSM with local kyphosis have been reported with mixed results. Baba et al. [16] reported that patients with preoperative kyphosis (mean angle of 11.7°) show significantly poorer neurological improvement. Suda et al. [11] also reported that outcomes of LP for CSM accompanying local kyphosis with an angle exceeding 13 degrees without signal changes and 5 degrees with signal changes in the cord were poorer than those for CSM without local kyphosis in their multivariate logistic regression analysis. In contrast, Kaptain et al. [17] and Uchida et al. [18] have shown that surgical outcomes were not correlated with preoperative cervical kyphosis. We speculated that their conclusion might have been due to the kyphosis in their patients being mild. In this study, cases preoperatively defined as K-line (-) showed poorer clinical outcome after LP compared to those with K-line ( +). Fujiyoshi et al. [10] was described that K-line was the measurement on lateral plain radiograph for cervical OPLL, however, in CSM patients, disc bulging was not able to be detected on such plain radiograph, therefore we used plain MRI for the K-line definition for CSM in the present study. K-line (-) patients were further divided into two groups as disc type and osseous type in MRI evaluation. The K-line drawn on the sagittal MRI has previously been reported to be a useful tool for predicting the surgical outcome for anterior cervical discectomy and fusion (ACDF) or LP for CSM [19]. The authors examined K-line on plain radiographs as well as did on MRIs. K-line ( +) and the osseous type of K-line (-) group which were classified from the MRI, and the classification on the plain radiograph were completely compatible to those on the MRI. In the disc type of K-line (-), six of the seven patients were classified as K-line ( +) on the radiograph because the disc bulging was not seen on the plain radiograph (data not shown). This might be the first report to observe preoperatively K-line (-) disc type changed to K-line ( +) due to resorption of the protruded disc during at the point of follow-up. These patients achieved better clinical outcomes compared to patients showing the osseous type, although it was still worse than those of K-line ( +) (without kyphosis at pre-operation). We speculated that the reason for this is the magnitude of remained kyphosis angle even after absorption of disc. Kasai and Uchida reported that the presence or absence of anterior or posterior subarachnoid space of the spinal cord in postoperative MRI correlated significantly with clinical outcome of LP [20]. Therefore, poor outcome of LP in the osseous type may result from incomplete, indirect anterior decompression of the spinal cord due to posterior shifting of the cord [21]. Miyamoto et al. [22] reported that posterior correction surgery for patients with CSM accompanied by local kyphosis resulted in a better clinical outcome than LP alone. Therefore, posterior correction surgery combined with LP thus is considered for the osseous type. With regards to the disc type, two surgical options are available: ACDF or PF combined with LP. In the present study, surgical outcome of disc type was still worse than of K-line ( +) group (without kyphosis). therefore, LP alone is not recommended due to the possibility that local kyphosis may deteriorate after LP [23, 24]. However, in the present study, the deterioration of cervical kyphosis was seen to be small (C2-7 angle; -6.0 ± 7.4 degrees at pre-operation and -6.7 ± 4.5 degrees at the follow-up, the disc type data). The limitation of the present study showed be noted. The submaterial of K-line (-) was small, in total 11 cases and comparing disc type with osseous type yields 7 and 4 cases respectively.

## Conclusion

The present study has indicated that K-line can be a tool to predict the clinical outcome of LP for CSM. The preoperative K-line (-) disc type can be changed to K-line ( +) during follow up with relatively better outcomes compared to the osseous type. However, the results were not satisfactory as K-line ( +) cases.

## Data Availability

The study was analyzed using data obtained from patients who provided informed consent. The datas used and/or analyzed in this study are available from the corresponding authour. Corresponding authour: Terumasa Ikeda, MD, E-mail: tikeda@med.kindai.ac.jp.

## Abbreviations

LP: Laminoplasty
OPLL: Posterior longitudinal ligament
CSM: Cervical spondylotic myelopathy
JOA: The Japanese orthopaedic association
RR: Recovery rate
Pre-op: Pre-operation
FU: Final follow-up

## References

[1] Hirabayashi K, Satomi K (1988). Operative procedure and result of expansive open-door laminoplasty. Spine (Phila Pa 1976).

[2] Hirabayashi K, Watanabe K, Wakano N, Suzuki N, Satomi K, Ishii Y (1983). Expansive open-door laminoplasty for cervical spinal stenotic myelopathy. Spine (Phila Pa 1976).

[3] Sodeyama T, Goto S, Mochizuki M, Takahashi J, Moriya H (1999). Effect of decompression enlargement laminoplasty for posterior shifting of the spinal cord. Spine (Phila pa 1976).

[4] Iwasaki M, Okuda S, Miyauchi A, Sakaura H, Mukai Y, Yonenobu K, Yoshikawa H (2007). Surgical strategy for cervical myelopathy due to ossification of the posterior longitudinal ligament: Part 1: Clinical results and limitations of laminoplasty. Spine (Phila pa 1976).

[5] Yamazaki A, Homma T, Uchiyama S, Katsumi Y, Okumura H (1999). Morphologic limitations of posterior decompression by midsagittal splitting method for myelopathy caused by ossification of the posterior longitudinal ligament in the cervical spine. Spine (Phila pa 1976).

[6] Baba H, Uchida K, Maezawa Y, Furusawa N, Azuchi M, Imura S (1996). Lordotic alignment and posterior migration of the spinal cord following en bloc open-door laminoplasty for cervical myelopathy: a magnetic resonance imaging study. J Neurol.

[7] Sakai K, Yoshii T, Arai Y, Hirai T, Torigoe I, Inose H, Tomori M, Sakaki K, Yuasa M, Yamada T, Matsukura Y, Oyaizu T, Morishita S, Okawa A. K-Line Tilt is a Predictor of Postoperative Kyphotic Deformity After Laminoplasty for Cervical Myelopathy Caused by Ossification of the Posterior Longitudinal Ligament. Global Spine J. 2021;21925682211012687. 10.1177/21925682211012687. 10.1177/21925682211012687 PMC10189327 33949218

[8] Rao H, Chen Y, Xu W, Zhou Z. Clinical Effects of Preoperative K-Line Tilt on Patient Outcomes After Laminoplasty for Cervical Ossification of the Posterior Longitudinal Ligament. World Neurosurg. 2021;150:e639-644. 10.1016/j.wneu.2021.03.071 33757888

[9] Xue R, Liu D, Li Y, Zhang D (2022). Different standing postures are the influencing factors for the efficacy of laminoplasty in the treatment of K-Line (-) patients with ossification of the posterior longitudinal ligament. Eur Spne J.

[10] Suda K, Abumi K, Ito M, Shono Y, Kaneda K, Fujiya M (2003). Local kyphosis reduces surgical outcomes of expansive open-door laminoplasty for cervical spondylotic myelopathy. Spine (Phila pa 1976).

[11] Fujiyoshi T, Yamazaki M, Kawabe J, Endou T, Furuya T, Koda M, Okawa A, Takahashi K, Konishi H (2008). A new concept for making decisions regarding the surgical approach for cervical ossification of the posterior longitudinal ligament: the K-line. Spine (Phila pa 1976).

[12] Chiba K, Toyama Y, Watanabe M, Maruiwa H, Matsumoto M, Hirabayashi K (2000). Impact of longitudinal distance of the cervical spine on the results of expansive open-door laminoplasty. Spine (Phila pa 1976).

[13] Miyazaki K, Tada K, Matsuda K, Okuno M, Yasuda T, Murakami H (1989). Posterior extensive simultaneous multisegument decompression with posterolateral fusion for cervical myelopathy with cervical instability and kyphotic and/or S-shaped deformities. Spine (Phila pa 1976).

[14] Tomita K, Kawahara N, Toribatake Y, Heller JG (1998). Expansive midline T-saw laminoplasty (modified spinous process-splitting) for the management of cervical myelopathy. Spine (Phila pa 1976).

[15] Japanese Orthopedic Association (1994). Scoring system for cervical myelopathy. J Jpn Orthop Assoc.

[16] Baba H, Maezawa Y, Furusawa N, imura S, Tomita K (1995). Flexibility and alignment of the cervical spine after laminoplasty for spondylotic myelopathy A radiographic study. Int Orthop.

[17] Kaptain GJ, Simmons NE, Replogle RE, Pobereskin L (2000). Incidence and outcome of kyphotic deformity following laminectomy for cervical spondylotic myelopathy. J Neurosurg.

[18] Uchida K, Nakajima H, Sato R, Yayama T, Mwaka ES, Kobayashi S, Baba H (2009). Cervical spondylotic myelopathy associated with kyphosis or sagittal sigmoid aliment: outcome after anterior or posterior decompression. J Neurosurg Spine.

[19] Hirai T, Yoshii T, Inose H, Yuasa M, Yamada T, Ushio S, Onuma H, Hirai K, Kobayashi Y, Utagawa K, Hashimoto J, Kawabata A, Sakai K, Kato T, Kawabata S, Okawa A (2019). Is Modified K-line a Powerful Tool of Surgical Decision Making for Patients With Cervical Spondylotic Myelopathy?. Clin Spine Surg.

[20] Kasai Y, Uchida A (2001). New evaluation method using preoperative magnetic resonance imaging for cervical spondylotic myelopathy. Arch Orthop Trauma Surg.

[21] Edwards CC 2nd and Heller JG. Cervical laminoplasty. Rothman-Simeone and Herkowitz's The Spine (fifth edition) Elsevier. 2006;877–895.

[22] Miyamoto H, Maeno K, Uno K, Kakutani K, Nishida K, Sumi M (2014). Outcomes of surgical intervention for cervical spondylotic myelopathy accompanying local kyphosis (comparison between laminoplasty alone and posterior reconstruction surgery using the screw-rod system). Eur Spine J.

[23] Sakaura H, Ohnishi A, Yamagishi A, Ohwada T (2019). Differences in Postoperative Changes of Cervical Sagittal Alignment and Balance After Laminoplasty Between Cervical Spondylotic Myelopathy and Cervical Ossification of the Posterior Longitudinal Ligament. Global spine J.

[24] Lee SH, Son DW, Lee JS, Sung SK, Lee SW, Song GS (2019). Does Extension Dysfunction Affect Postoperative Loss of Cervical Lordosis in Patients Who Undergo Laminoplasty?. Spine (Phila pa 1976).

